# Improved Synthesis of 5-Substituted 1*H*-Tetrazoles via the [3+2] Cycloaddition of Nitriles and Sodium Azide Catalyzed by Silica Sulfuric Acid

**DOI:** 10.3390/ijms13044696

**Published:** 2012-04-12

**Authors:** Zhenting Du, Changmei Si, Youqiang Li, Yin Wang, Jing Lu

**Affiliations:** 1 College of Science, Northwest A & F University, Yangling, Shaanxi 712100, China; E-Mails: dootritiger@yahoo.com.cn (C.S.); lyq812322926@163.com (Y.L.); whuhu@yahoo.cn (Y.W.); chinalulu139@126.com (J.L.); 2 Key Laboratory of Protection and Utilization of Biological Resources in Tarim Basin, Talimu University, Alaer, Xinjiang 843300, China

**Keywords:** silica sulfuric acid, 5-substituted 1*H*-tetrazoles, [3+2] cycloaddition

## Abstract

A silica supported sulfuric acid catalyzed [3+2] cycloaddition of nitriles and sodium azide to form 5-substituted 1*H*-tetrazoles is described. The protocol can provide a series of 5-substituted 1*H*-tetrazoles using silica sulfuric acid from nitriles and sodium azide in DMF in 72%–95% yield.

## 1. Introduction

In recent years, the growth of the tetrazole chemistry has been significant [1,2], mainly as a result of the central roles played by tetrazoles in coordination chemistry as nitrogen-containing heterocyclic ligands [3], in materials applications as specialty explosives, information recording systems, rocket propellants and in agrichemical applications [4,5]. In particular, tetrazoles can be used as equivalent replacements for carboxylic moiety in drug design, with the advantage over carboxylic moieties being that they are resistant to many biological metabolic degradation pathways [6]. In fact, several leading compounds have been synthesized and tested for pharmaceutical purposes [7–9]. Furthermore, tetrazole moieties can be used as important synthons in synthetic organic chemistry due to their characteristic electronic property [10–12].

The proton acid-catalyzed cycloaddition between hydrazoic acid and nitriles has long been one of the main routes to 5-substituted tetrazoles. However, this standard procedure suffers a dangerous potential explosion with large excess amounts of harmful hydrazoic acid [13]. Consequently, it is urgent to improve the synthetic method of obtaining 5-substituted 1*H*-tetrazoles. A number of catalytic systems of [3+2] reaction of sodium azide and nitriles were reported by various research teams, such as Zn(II) salts [14–16], AlCl_3_ [17], Et_3_N·HCl [18], BF_3_·OEt_2_ [19], TBAF [20], Pd(PPh_3_)_4_ [21], Zn/Al hydrotalcite [22], ZnO [23], Zn-Cu alloy [24], Cu_2_O [25] and FeCl_3_-SiO_2_ [26]. The limitations of the existing protocols realized in terms of longer reaction time, stringent conditions, expensive and toxic metal catalysts (e.g., Pd(PPh_3_)_4_ is costly and air sensitive), tedious work-ups and unable or unsatisfactory recovery of catalyst. Therefore, it is necessary to develop a more efficient and convenient method that avoids these drawbacks and could be used both on a laboratory and industrial scale.

Meanwhile, recyclable and efficient heterogeneous catalysts have attracted vastly soaring interest in the context of appealing to green synthesis. As a case in point, silica sulfuric acid is cheapest and easiest to be implanted for industrial use [27,28]. Because of its unique chemical and physical properties, silica sulfuric acid has several advantages such as nonvolatility, adjustable acidity, ease of handling and environmentally safe disposal. The erosion of equipment will be dramatically reduced when it is used as a substituent of traditional protonic acid in industry [29]. We are interested in an efficient and convenient formation of 5-substituted 1*H*-tetrazoles through nitrile and sodium azide catalyzed by silica sulfuric acid. To the best of our knowledge, there is no report on any solid acid catalytic synthesis of 5-substituted 1*H*-tetrazoles from nitrile and sodium azide. Herein, we wish to report a facile synthesis of 5-substituted 1*H*-tetrazoles catalyzed by silica sulfuric acid in 72–95% yield.

## 2. Results and Discussion

To begin with, the silica sulfuric acid was prepared according to Shaterian’s method [28] and the amount of H^+^ in silica sulfuric acid was titrated and calculated (0.05 g of silica sulfuric acid equal to 0.1 mmol). The solvents were screened and the result was shown in Table 1. Our studies subsequently showed that the nature of reaction solvents was extremely important for this reaction. Obviously, alcohols (Table 1, entries 1–2) were not suitable for this reaction. Low polar solvents such as toluene (entry 4) and chloroform (entry 5) both give unsatisfactory yield. Both DMF (entry 5) and DMSO (entry 6) gave excellent yields, therefore, DMF was chosen as the most suitable solvent because of its easier workup compared with DMSO.

Subsequently, the effect of catalyst loading was investigated. To our interest, for the model reaction of benzonitriles and sodium azide, 100% mol catalyst is enough to perform cyclization. Lower catalyst loading (50%) would lead to longer reaction time and lower yield (Table 1, entry 7) and higher ratio catalyst (200%) only gave a slightly increase of yield (Table 1, entry 8). Finally, we set up the optimized reaction conditions that are DMF as solvent, 100% molar ratio silica sulfuric acid as catalyst, at refluxing temperature (Table 1, entry 5).

**Figure 1:**
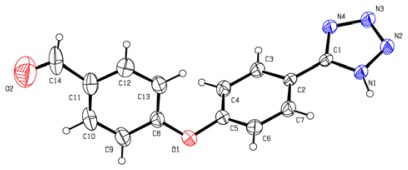
ORTP drawing of 10.

With the optimized reaction conditions, we next examined the scope of silica sulfuric acid catalyzed cyclization for the synthesis of 5-substituted 1*H*-tetrazoles. The results are summarized in Table 2. A wide range of structurally diverse nitriles (Table 2), including aromatic (Table 2, entries 1–2, 5–7, 10) and aliphatic nitriles (Table 2, entries 3, 8–9, 12) were subjected under this protocol to provide the corresponding 5-substituted 1*H*-tetrazoles in high yields. Neither the electronic nature nor the satiric hindrance of the substitution at the both aromatic rings had any obvious influence upon the reactivity. All the products in our reactions listed in Table 2 were easily characterized on the basis of physical and spectral data and also by comparison with authentic samples or reported ones. The structure of product of entry 10 was determined by X-ray crystallography and the ORTEP was shown in Figure 1 [30].

In addition, we investigated the reusability and recycling of silica sulfuric acid. As to the model reaction, the catalyst was separated by simple filtration after completion of reaction. The recovered silica sulfuric acid was reused directly three times without significant decrease in activity.

## 3. Experimental Section

The IR spectra were recorded on a Perkin-Elmer 2000 FTIR spectrometer. ^1^H and ^13^C NMR data were recorded in DMSO with Bruker-AM 500 unless noted otherwise. The chemical shifts were reported in ppm relative to TMS. Mass spectra were recorded on a Thermo Fisher mass spectrometer by electrospray ionization method (ESI). Column chromatography were generally performed on silica gel (200–300 mesh) eluting with petroleum ether:EtOAc (20:1–1:1 v/v) and TLC inspections on silica gel GF254 plates with petroleum ether:EtOAc (20:1–1:1 v/v) unless noted otherwise.

General procedure for the preparation of through nitriles and sodium azide catalyzed by silica sulfuric: A suspension of nitriles (1 mmol), sodium azide (1.2 mmol) and silica sulfuric acid (500 mg, 1 mmol) in DMF (10 mmol) was heated to reflux for 4–12 hours with stirring. After the completion of the reaction, the precipitate of solid acid was filtered and washed, the filtrate was evaporated under vacuum and the crude product was purified by recrystallization or column chromatography on silica gel eluting with a mixture of petroleum ether and ethyl acetate to give 5-substituted 1*H*-tetrazoles.

*5-Phenyl-1H-tetrazole* **1**: white needles, m.p. 215–216 °C. ^1^H NMR (500 Hz, DMSO-*d*_6_): 8.05 (s, 2 H, Ar-H), 7.61 (s, 3 H, Ar-H) ppm. ^13^C NMR (125 Hz, DMSO-d_6_): 131.7, 129.9, 127.4, 124.6 ppm. ESI-MS (*m/z*): M-H = 145. IR: 1485, 1564, 1609, 2916 cm^−1^.

*5-(2-Chlorophenyl)-1H-tetrazole* **2**: yellowish solid, m.p. 180–181 °C. ^1^H NMR (500 Hz, DMSO-*d*_6_): 7.83 (s, 1 H, Ar-H), 7.72 (s, 1 H, Ar-H), 7.65 (s, 1 H, Ar-H), 7.58 (s, 1 H, Ar-H) ppm. ^13^C NMR (125 Hz, DMSO-*d*_6_): 133.1, 132.4, 132.2, 130.9, 128.3, 124.6 ppm. ESI-MS *m/z* 179 [M – H]^−^. IR: 1470, 1563, 1602, 2923 cm^−1^.

*5-(4-Bromobenzyl)-1H-tetrazole* **3**: white needles, m.p. 178–180 °C. ^1^H NMR (500 Hz, DMSO-*d*_6_): 7.57 (s, 2 H, Ar-H), 7.28 (s, 2 H, Ar-H), 4.32 (s, 2 H, CH_2_–H) ppm. ^13^C NMR (125 Hz, DMSO-*d*_6_): 136.3, 132.6, 132.5, 131.9, 121.2, 29.2. ESI-MS *m/z* 238 [M – H]^−^. IR: 1489, 1584, 1660, 2848 cm^−1^.

*5-(4-Bromophenyl)-1H-tetrazole* **4**: yellowish solid, m.p. 268–270 °C (decompose). ^1^H NMR (500 Hz, DMSO-*d*_6_): 17.00 (brs, 1 H, N-H), 8.01–7.98 (m, 2 H, Ar-H), 7.85–7.82 (m, 2 H, Ar-H) ppm. ^13^C NMR (125 Hz, DMSO-*d*_6_): 155.0, 132.5, 128.9, 124.7, 123.6 ppm. ESI-MS *m/z* 224 [M – H]^−^. IR: 1482, 1561, 1604, 2850 cm^−1^.

*5-(4-Fluorophenyl)-1H-tetrazole* **5**: yellowish solid, m.p. 114–116 °C. ^1^H NMR (500 Hz, DMSO-*d*_6_): 16.91 (brs, 1 H, N-H), 8.12–8.07 (m, 2 H, Ar-H), 7.50–7.45 (m, 2 H, Ar-H) ppm. ^13^C NMR (125 Hz, DMSO-*d*_6_): 163.6 (d, *J* = 249 Hz), 154.6, 129.5 (d, *J* = 8.9 Hz), 116.6 (d, *J* = 22.3 Hz) ppm. ESI-MS *m/z* 163 [M – H]^−^. IR: 1505, 1610, 2991 cm^−1^.

*5-(3-Methoxyphenyl)-1H-tetrazole* **6**: white solid, m.p. 156–158 °C. ^1^H NMR (500 Hz, DMSO-*d*_6_): 16.88 (brs, 1 H, N-H), 7.64–7.62 (m, 1 H, Ar-H),7.60–7.59 (m, 1 H, Ar-H), 7.53 (t, 1 H, *J* = 8.05 Hz, Ar-H), 7.17 (ddd, 1 H, *J* = 0.8, 2.55, 3.4 Hz, Ar-H), 3.86 (s, 3 H, CH_3_-H) ppm. ^13^C NMR (125 Hz, DMSO-*d*_6_): 159.7, 155.0, 130.6, 125.2, 119.1, 117.0, 112.0, 55.3 ppm. ESI-MS *m/z* 175 [M – H]^−^. IR: 1490, 1564, 1711, 2843 cm^−1^.

*5-(2-Bromophenyl)-1H-tetrazole* **7**: yellowish solid, m.p. 178–179 °C. ^1^H NMR (500 Hz, DMSO-*d*_6_): 16.92 (brs, 1 H, N-H), 7.88 (dd, 1 H, *J* = 1.2, 1.3 Hz, Ar-H), 7.72 (dd, 1 H, *J* = 1.8, 1.8 Hz, Ar-H), 7.60 (td, 1 H, *J* = 1.3, 7.45 Hz, Ar-H), 7.55 (td, 1 H, *J* = 1.9, 7.85 Hz, Ar-H) ppm. ^13^C NMR (125 Hz, DMSO-*d*_6_): 154.6, 133.5, 132.7, 132.0, 128.1, 126.4, 121.7 ppm. ESI-MS *m/z* 224 [M – H]^−^. IR: 1476, 1574, 1604 cm^−1^.

*5-Benzyl-1H-tetrazole* **8**: white solid, m.p. 118–120 °C. ^1^H NMR (500 Hz, DMSO-*d*_6_): 16.18 (brs, 1 H, N-H), 7.35–7.32 (m, 2 H, Ar-H), 7.28–7.25 (m, 3 H, Ar-H), 4.29 (s, 2 H, CH_2_-H) ppm. ^13^C NMR (125 Hz, DMSO-*d*_6_): 155.2, 135.9, 128.7, 128.6, 127.0, 28.9 ppm. ESI-MS *m/z* 159 [M – H]^−^. IR: 1493, 1531, 1548, 2951 cm^−1^.

*5-(4-Methoxybenzyl)-1H-tetrazole* **9**: yellowish solid, m.p. 154–156 °C. ^1^H NMR (500 Hz, DMSO-*d*_6_): 16.07 (brs, 1 H, N-H), 7.20–7.18 (m, 2 H, Ar-H), 6.91–6.88 (m, 2 H, Ar-H), 4.20 (s, 2 H, CH_2_-H), 3.70 (s, 3 H, CH_3_-H) ppm. ^13^C NMR (125 Hz, DMSO-*d*_6_): 158.3, 129.7, 114.1, 55.1, 28.0 ppm. ESI-MS *m/z* 189 [M – H]^−^. IR: 1513, 1554, 1612, 2838 cm^−1^.

*4-(4-(1H-Tetrazol-5-yl)phenoxy)benzaldehyde* **10**: yellow solid, m.p. 172–174 °C. ^1^H NMR (500 Hz, DMSO-*d*_6_): 9.97 (s, 1 H, CHO-H), 8.13 (d, 2 H, *J* = 9.0 Hz, Ar-H), 7.98 (d, 2 H, *J* = 8.5 Hz, Ar-H), 7.37 (d, 2 H, *J* = 8.5 Hz, Ar-H), 7.26 (d, 1 H, *J* = 8.5 Hz, Ar-H) ppm. ^13^C NMR (125 Hz, DMSO-*d*_6_): 192.1, 161.7, 157.9, 132.6, 132.5, 129.8, 120.9, 119.1, 119.0, 107.2 ppm. ESI-MS *m/z* 265 [M – H]^−^. IR: 1496, 1596, 1616, 1700 cm^−1^.

*3-(1H-Tetrazol-5-yl)-2H-chromen-2-one* **11**: greenish solid, m.p. 244–246 °C. ^1^H NMR (500 Hz, DMSO-*d*_6_): 9.05 (s, 1 H, CH-H), 8.02 (d, 1 H, *J* = 7.5 Hz, Ar-H), 7.80–7.76 (m, 1 H, Ar-H), 7.56 (d, 1 H, *J* = 8.0 Hz, Ar-H), 7.53 (t, 1 H, *J* = 7.5 Hz, Ar-H) ppm. ^13^C NMR (125 Hz, DMSO-*d*_6_): 158.6, 154.1, 144.9, 134.5, 130.5, 125.7, 118.9, 116.9, 112.8, 102.2 ppm. ESI-MS *m/z* 213 [M – H]^−^. IR: 1577, 1612, 1697, 3299 cm^−1^.

*5-(4-Nitrobenzyl)-1H-tetrazole* **12**: Yellowish needles, m.p. 188–190 °C. ^1^H NMR (500 Hz, DMSO-*d*_6_): 8.23 (d, 2 H, *J* = 8.5 Hz, Ar-H), 7.58 (d, 2 H, *J* = 8.5 Hz, Ar-H), 4.50 (s, 2 H, CH_2_-H) ppm. ^13^C NMR (125 Hz, DMSO-*d*_6_): 156.1, 147.1, 144.2, 130.7, 124.3, 29.2 ppm. ESI-MS *m/z* 204 [M – H]^−^. IR: 1349, 1536, 1584, 2719 cm^−1^.

## 4. Conclusions

In conclusion, we have described herein silica sulfuric acid catalyzed highly efficient, one-pot, protocol for the synthesis of 5-substituted 1*H*-tetrazoles through the [3+2] cycloaddition of various nitriles and sodium and azide in refluxing DMF in excellent yields. This method provides high conversions and yields, simplicity in operation and cost-effectiveness. Thus, we believe that this novel methodology will be a practical alternative to the existing procedures to cater to the needs of academia as well as industries. Further work is in progress to broaden the scope of this practical process.

## Figures and Tables

**Table 1:** SiO_2_-H_2_SO_4_ catalyzed [3+2] cycloaddition of benzonitriles and sodium azide in different solvents.

Entry	Solvent	Time	mol Ratio of SiO_2_-H_2_SO_4_	Yield (100%)
**1**	Methanol	12	50%	<10
**2**	Ethanol	12	50%	10
**3**	Toulene	12	50%	5
**4**	Chloroform	12	50%	No reaction
**5**	DMF	5	50%	92
**6**	DMSO	5	50%	89
**7**	DMF	10	50%	85
**8**	DMF	5	200%	93

**Table 2:** Silica sulfuric acid catalyzed synthesis of 5-substituted 1*H*-tetrazoles through [3+2] cycloaddition of benzonitriles and sodium azide in DMF.

Entry	Nitriles	Tetrazoles	Yield (%)
**1**	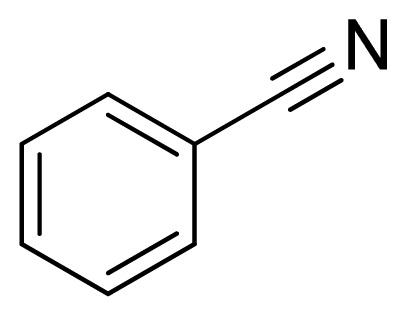	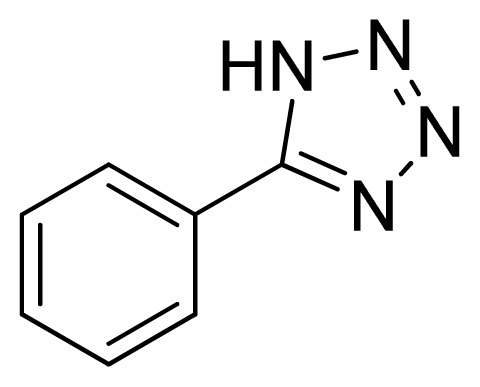	88
**2**	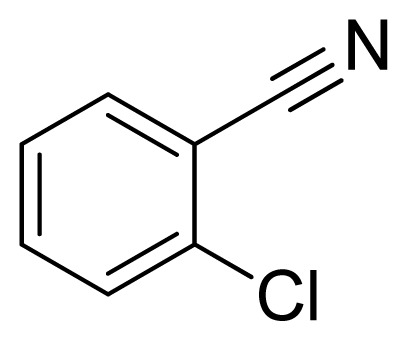	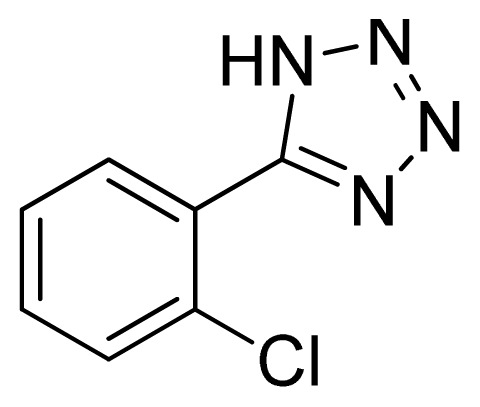	72
**3**	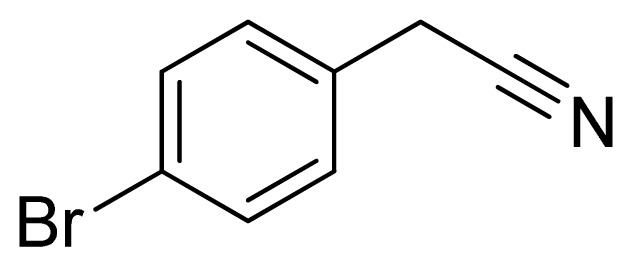	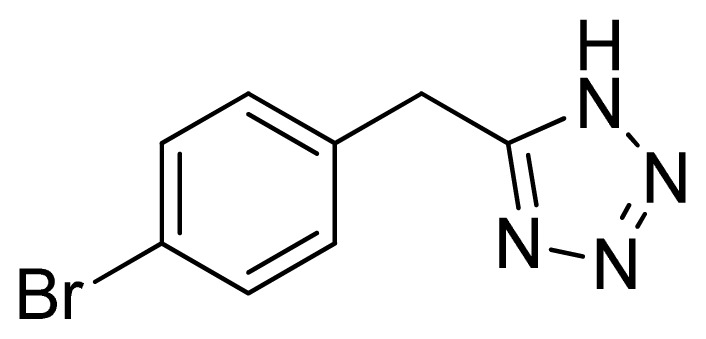	88
**4**	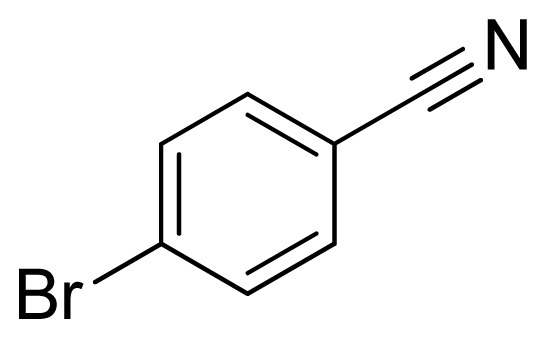	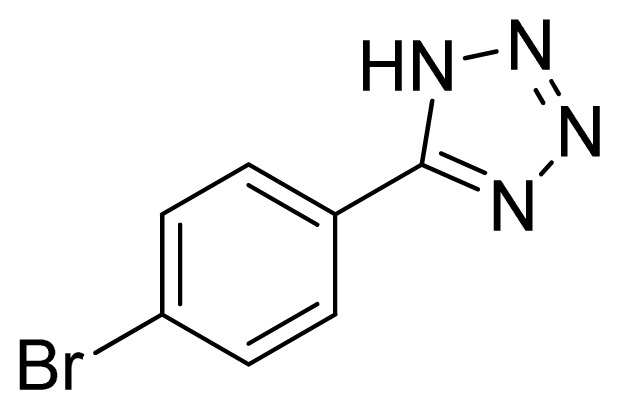	79
**5**	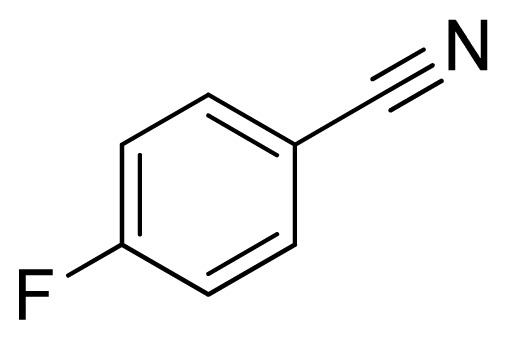	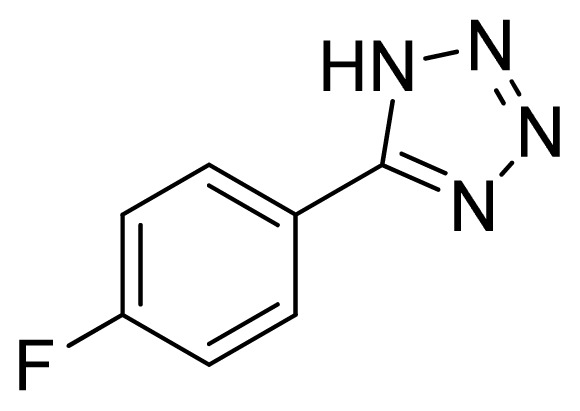	88
**6**	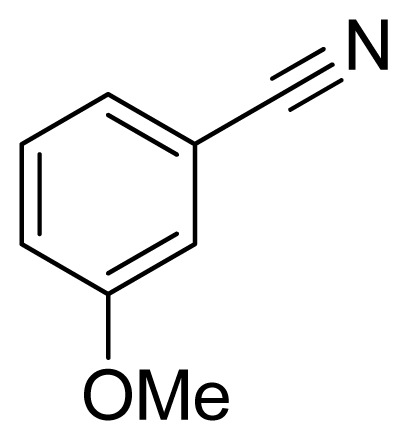	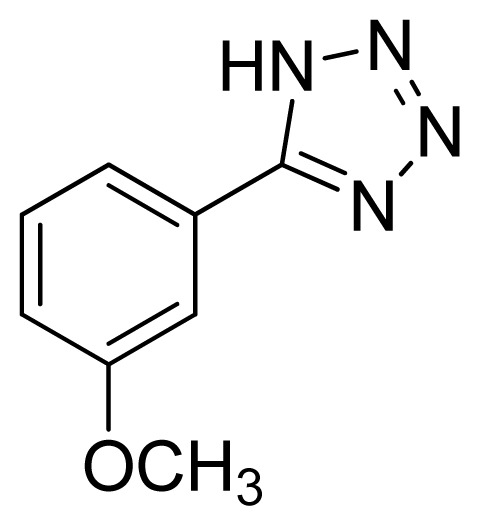	95
**7**	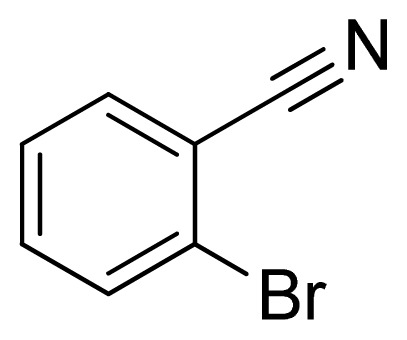	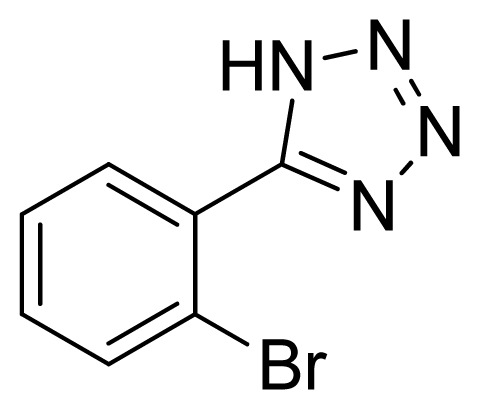	92
**8**	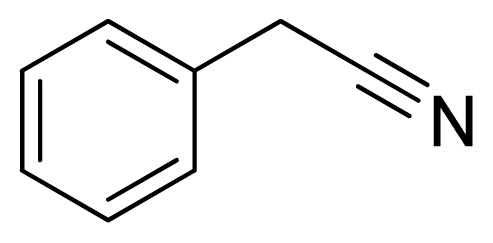	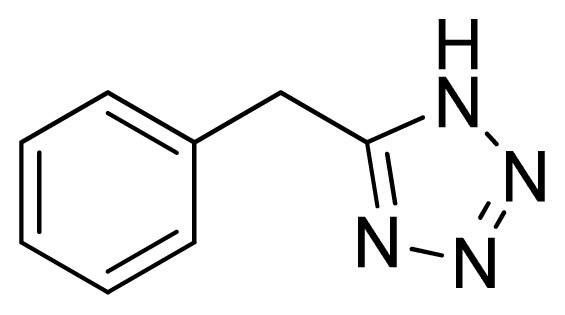	74
**9**	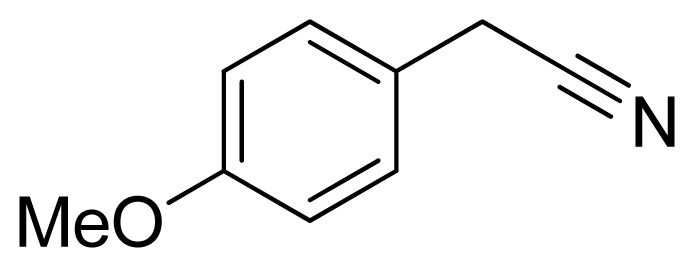	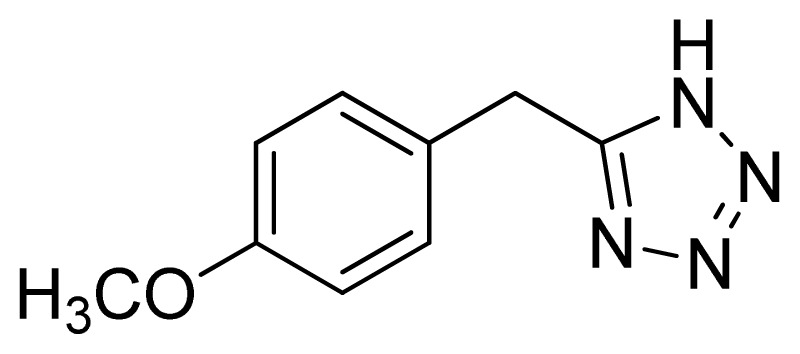	72
**10**	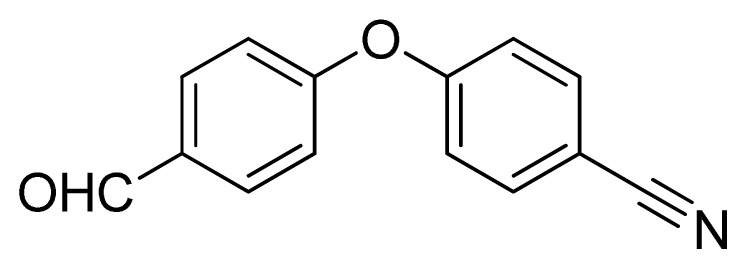	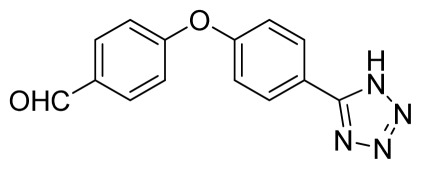	88
**11**	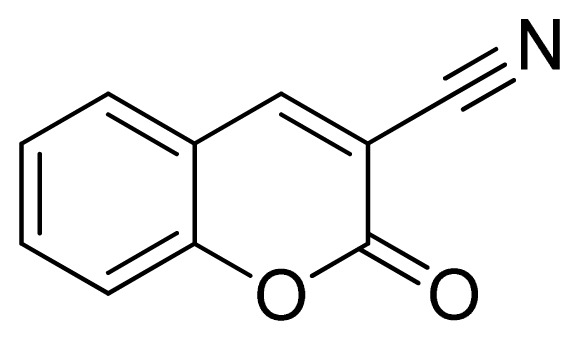	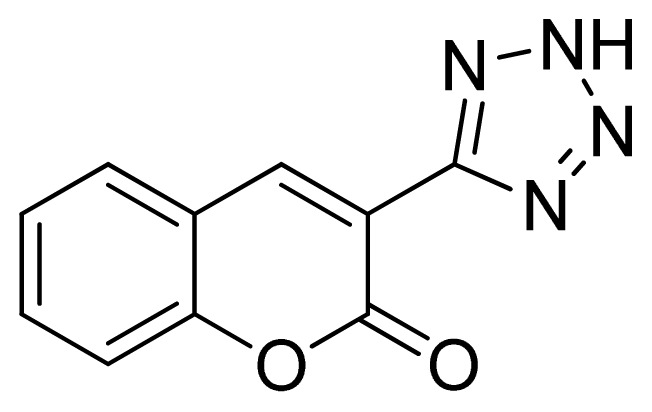	80
**12**	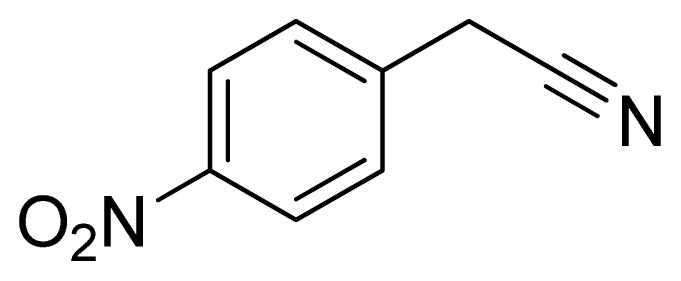	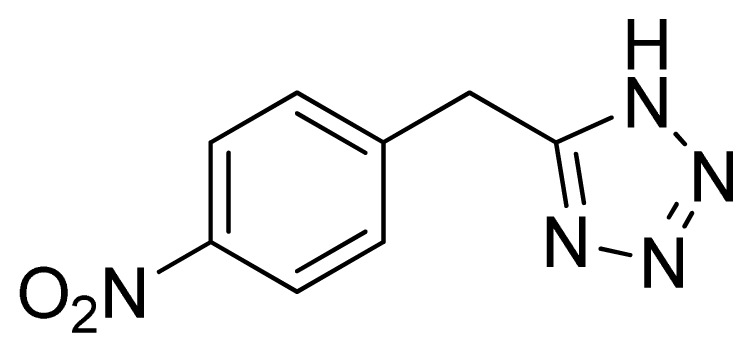	76
